# Factors associated with type 1 gastric neuroendocrine tumor occurrence in autoimmune atrophic gastritis: insights from a real-world cohort

**DOI:** 10.3389/fendo.2026.1880679

**Published:** 2026-07-07

**Authors:** Roberta Elisa Rossi, Matteo Ferraris, Lorenzo Petronio, Benedetta Masoni, Luca Di Stefano, Alexia Francesca Bertuzzi, Sara Fraticelli, Andrea Gerardo Antonio Lania, Alessandro Zerbi, Cesare Hassan, Alessandro Repici

**Affiliations:** 1 Gastroenterology and Endoscopy Unit, IRCCS Humanitas Research Hospital, Milan, Italy; 2 Department of Biomedical Sciences, Humanitas University, Milan, Italy; 3 Hematology and Oncology, Scientific Institute for Research, Hospitalization and Healthcare (IRCCS) Humanitas Research Hospital, Milan, Italy; 4 Pathology Service, IRCCS, Humanitas Research Hospital, Milan, Italy; 5 Endocrinology and Diabetology Unit, IRCCS Humanitas Research Hospital, Milan, Italy; 6 Pancreatic Surgery Unit, IRCCS Humanitas Research Hospital, Milan, Italy

**Keywords:** associated factors, autoimmune atrophic gastritis, gastric neuroendocrine tumors, occurrence, risk factors

## Abstract

**Background:**

Autoimmune gastritis (AIG) is characterized by chronic inflammation and represents a key step in the multistage precancerous process of the stomach. Patients with AIG are at increased risk of developing type I gastric neuroendocrine tumors (T1gNETs), but clinical and histological associated factors still remain incompletely defined.

**Aim:**

To evaluate the occurrence of T1gNETs in our AIG patients cohort and to identify clinical, biochemical, and histological factors associated with tumor development.

**Methods:**

Retrospective analysis of histologically confirmed AIG followed at Humanitas Research Hospital (from January 2020 to February 2025). Clinical, biochemical, and histological variables were extracted from a dedicated database. Associations between T1gNETs incidence and risk factors (smoking, BMI, gastrin,chromogranin- CgA levels, histology, clinical presentation) were tested using chi-square, Spearman and Mann-Whitney tests, with bootstrap-derived confidence intervals for effect sizes.

**Results:**

Among 175 AIG patients, 28 (16%) were diagnosed with T1gNETs. ECL cell hyperplasia emerged as the strongest histological factor associated with T1gNETs (82.1 vs 55.8; p= 0.0109, OR = 3.65). Conversely, dyspeptic symptoms showed a significant inverse correlation with tumor occurrence (21.4% vs 43.5%; p=0.035, OR = 0.35), suggesting that T1gNETs often develop in asymptomatic patients. No significant predictive value was found for smoking, alcohol, BMI, severe hypergastrinemia/CgA (>3x ULN) levels or degree of corpus atrophy, although a trend was observed for corpus metaplasia.

**Conclusions:**

Patients with AIG face significantly elevated occurrence of T1gNETs compared with the general population, with percentage being in line with recent studies. ECL hyperplasia was found as a factor associated with NET development, according to literature, confirming that the chronic stimulation of a cell with the ability to proliferate, results in tumors formation. Most patients with T1gNETs were asymptomatic and no correlation was found with smoking, alcohol and BMI. As a future perspective, these findings, even though preliminary, may help in the risk stratification of patients for more personalized endoscopic surveillance protocols, but further data are warranted to better standardize their use.

## 1 Introduction

Autoimmune gastritis (AIG) is a chronic, immune-mediated inflammatory disease, characterized by the immune-mediated destruction of gastric parietal cells and progressive mucosal atrophy driven by the autoreactive CD4+ T-cell response against the gastric H+/K+ ATPase (1). Parietal cells are damaged or destroyed and gastric acid is reduced; this condition leads to hypo- or achlorhydria and subsequent high circulating gastrin levels. Enterochromaffin like cells (ECL) respond to gastrin by releasing histamine, which in turn enhances gastric acid secretion. This circle triggers ECL-cells hyperplasia and predispose to type I gastric neuroendocrine tumors (T1gNETs) (2–4). This condition is tipically associated with female sex and older age, similarly to other autoimmune disorders, with a reported female-to-male ratio of 2–3:1 and a median age at diagnosis of approximately 60 years (5, 6).

Gastric neuroendocrine tumors (gNETs) are rare neoplasms arising from enterochromaffin-like (ECL) cells and are classified into five subtypes, of which type 1 (T1gNETs) is the most common and is typically associated with AIG (7). T1gNETs account for approximately 70–80% of all gNETs, are typically diagnosed in adults with a median age of 60–70 years, and are more common in women, likely reflecting the underlying autoimmune background (8, 9). The risk of developing T1gNETs in the natural history of AIG patients is well established, affecting 4-12% of AIG patients lifelong (10), which, together with the risk of developing gastric adenocarcinoma, represents the reason why the European Society of Gastrointestinal Endoscopy (ESGE) recommend high-quality endoscopic follow-up every 3 years in AIG patients (11). Presence of atrophy, metaplasia and ECL hyperplasia are well established risk factors (11–13) serum biomarkers like chromogranin A (CgA) and gastrin are used in the AIG setting, but their ability to predict gastric lesions is inconclusive (13, 14).

Although the pathogenetic role of hypergastrinemia and ECL-cell hyperplasia is well established, the relative contribution of clinical, biochemical and histological variables to T1gNET occurrence remains incompletely characterized (15).

The aim of current study was to assess the association between T1gNETs occurrence and potential clinical (i.e., Body Mass Index-BMI, smoking, alcohol, presence of dyspeptic symptoms), biochemical (i.e., chromogranin A-CgA and gastrin levels) or histological associated factors.

## 2 Materials and methods

### 2.1 Study design

This was a single-center retrospective observational study conducted at the Gastroenterology and Endoscopy Unit of IRCCS Humanitas Research Hospital, Rozzano, Milan, Italy. All consecutive patients with a histologically confirmed diagnosis of autoimmune atrophic gastritis (AIG) evaluated between January 2020 and February 2025 were screened for eligibility. The diagnosis of AIG was established histologically based on the presence of corpus-restricted gastric atrophy and/or intestinal metaplasia, and confirmed by serological markers, including hypergastrinemia and/or positivity for anti-parietal cell antibodies (APCA). All endoscopic procedures were performed using high-definition white-light endoscopy; biopsies were collected according to the Updated Sydney System.

Clinical, biochemical, and histological data were retrieved from institutional electronic medical records and from a dedicated clinical database used for the follow-up of patients with AIG. Inclusion criteria were adult patients (≥18 years) with histological confirmation of AIG and availability complete clinical, biochemical, and histological data. Exclusion criteria included atrophic gastritis of non-autoimmune etiology and incomplete clinical or histological data. The primary outcome of the study was the occurrence of T1gNETs during follow-up. The diagnosis of T1gNET was established on the basis of histological evaluation of endoscopic biopsies or resected lesions according to current pathological criteria (6).

Clinical variables collected included demographic characteristics, smoking status, alcohol consumption, BMI, and the presence of dyspeptic symptoms at clinical evaluation. Dyspeptic symptoms were defined according to Rome IV criteria as the presence of postprandial fullness, early satiety, epigastric pain, or epigastric burning for at least three months, with symptom onset at least six months before diagnosis, and were further classified into postprandial distress syndrome (PDS) and epigastric pain syndrome (EPS) T1gNET was established on the basis of histological evaluation of endoscopic biopsies or resected lesions according to current pathological criteria (8).

### 2.2 Clinical, biochemical, and histological assessment

All patients underwent standardized clinical and endoscopic evaluation as part of routine management of autoimmune gastritis.

Anthropometric data included BMI, while lifestyle factors such as smoking status and alcohol consumption were also recorded.

Biochemical evaluation included fasting serum gastrin and CgA levels. These markers were classified as normal or elevated according to laboratory reference values.

All patients underwent upper gastrointestinal endoscopy with systematic biopsy sampling of the gastric antrum and corpus according to the Updated Sydney System (16).

Histological examination assessed the presence and severity of gastric atrophy, intestinal metaplasia, and ECL cell hyperplasia. Gastric atrophy was evaluated separately in the antrum and in the corpus–fundus and graded as absent, mild, moderate, or severe according to the Updated Sydney System. Intestinal metaplasia and ECL cell hyperplasia were recorded as present or absent.

The occurrence of T1gNETs during follow-up was identified through endoscopic detection and histological confirmation. Informed consent was waived, given the use of retrospective historic de-identified data.

### 2.3 Statistical analysis

Statistical analysis was performed using the clinical dataset including demographic, clinical, biochemical, and histological variables.

Descriptive statistics were used to summarize baseline characteristics of the study population. Continuous variables were reported as mean ± standard deviation (SD), while categorical variables were expressed as frequencies and percentages.

Patients were stratified into two groups according to the occurrence of type 1 gastric neuroendocrine tumors (T1gNETs): patients who developed T1gNETs and patients who did not.

Comparisons between groups were performed using appropriate statistical tests. Continuous variables were compared using the Mann–Whitney U test. Associations between categorical variables and T1gNET occurrence were assessed using Fisher’s exact test.

Odds ratios (OR) with corresponding 95% confidence intervals (CI) were calculated to estimate the strength of association between potential risk factors and the development of T1gNETs. When zero cells were observed in contingency tables, a continuity correction of 0.5 (Haldane–Anscombe correction) was applied for OR calculation.

Given the limited number of outcome events (n=28), multivariable logistic regression analysis was not performed to avoid model overfitting, in accordance with the events-per-variable criterion.

All statistical tests were two-tailed and a p-value <0.05 was considered statistically significant.

## 3 Results

### 3.1 Cohort characteristics

During the study period, a total of 197 patients were referred to our out-patient clinic; of these, 22 patients were excluded due to insufficient data. Therefore, 175 patients with histologically confirmed AIG were included in the final analysis, 130 females (74.3%) and 45 males (25.7%), with a median age at diagnosis of 56 years old (range: 17–88 years) and a median follow up of 28 months (range: 0 to 144).

**Figure 1:**
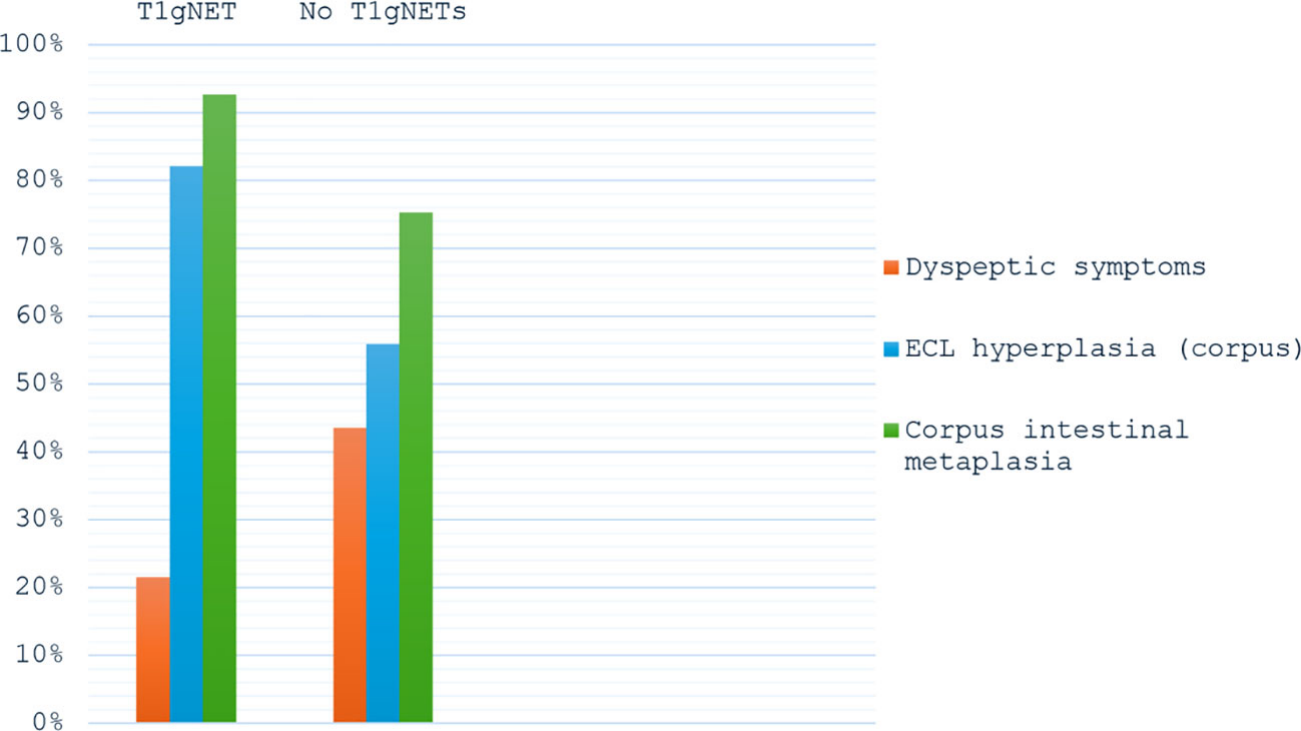
Bar chart showing the percentage of selected clinical and histological variables in patients with and without type 1 gastric neuroendocrine tumors (T1gNETs).

**Table 1** summarizes the baseline characteristics of the study population. The occurrence of T1gNETs was observed in 28 patients (16%) during follow-up. Among these, 19 were female (67.9%) and 9 were male (32.1%), with a median age of 57.5 years (range: 43–72 years). Notably, in 18 out of 28 patients (64.3%) who developed T1gNETs, the diagnosis of autoimmune atrophic gastritis and T1gNET was established during the same endoscopic assessment (**Table 2**; **Figure 1**).

**Table 1:** Baseline characteristics of the study population (n = 175).

Variable	Overall (n=175)
Age at diagnosis, years, median (range)	56 (17-88)
Sex, n (%)	
Female	130 (74.3%)
Male	45 (25.7%)
Current smokers, n (%)	19 (10.86%)
Alcohol consumption, n (%)	35 (20%)
Dyspeptic symptoms, n (%)	70 (40%)
*Helicobacter pylori* infection, n (%)	13 (7.4%)
Autoimmune comorbidities, n (%)	89 (50.8%)

**Table 2:** Comparison between patients with and without type 1 gastric neuroendocrine tumors (T1gNETs).

Variable	T1gNETs (n = 28)	No T1gNETs (n = 147)	p-value	OR (95% CI)
Demographics				
Age at diagnosis, years, mean ± SD	57.5 ± 14.5	56.3 ± 14.1	0,6256	-–-
BMI, kg/m², mean ± SD	25.4 ± 4.0	24.4 ± 4.5	0,2302	-–-
Male sex, n (%)	9 (32.1%)	36 (24.7%)	0,48	1.45 (0.60–3.48)
Lifestyle factors				
Current smokers, n (%)	1 (3.7%)	18 (12.3%)	0,3148	0.27 (0.03–2.14)
Alcohol consumption, n (%)	6 (22.2%)	29 (19.9%)	0,796	1.15 (0.43–3.12)
Clinical presentation				
Dyspeptic symptoms, n (%)	6 (21.4%)	64 (43.5%)	0,035	0.35 (0.14–0.92)
Helicobacter pylori infection, n (%)	1 (3.6%)	12 (8.1%)	0,6954	0.60 (0.10–3.40)
Autoimmune comorbidities, n (%)	14 (50.0%)	75 (51.0%)	1	0.96 (0.43–2.13)
Biochemical markers				
Elevated gastrin (>3× ULN), n (%)	13/16 (81.2%)	29/46 (63.0%)	0,2261	2.54 (0.63–10.21)
Elevated CgA, n (%)	11/15 (73.3%)	21/39 (53.8%)	0,2302	2.36 (0.64–8.70)
Histological findings				
ECL hyperplasia (corpus), n (%)	23 (82.1%)	82 (55.8%)	0,0109	3.65 (1.31–10.12)
ECL hyperplasia (antrum), n (%)	4 (14.3%)	13 (8.8%)	0,4824	1.72 (0.52–5.71)
Corpus atrophy (any grade), n (%)	24 (85.7%)	130 (88.4%)	0,7506	0.73 (0.24–2.24)
Antral atrophy (any grade), n (%)	10 (35.7%)	55 (37.4%)	1	0.95 (0.41–2.16)
Corpus intestinal metaplasia, n (%)	25/27 (92.6%)	110/146 (75.3%)	0,0732	3.37 (0.87–13.02)
Antral intestinal metaplasia, n(%)	7/27 (25.9%)	30/145 (20.7%)	0,6104	1.39 (0.55–3.50)

### 3.2 Characteristics, management and outcomes of T1gNETs

Among the 28 patients diagnosed with T1gNETs, 20 (71.4%) presented with a single lesion, whereas 8 (28.6%) had multifocal disease. Tumor size was available for 26 patients, while data were unavailable for 2 patients who had been diagnosed and managed in external institutions. The median lesion size was 4.5 mm (range 2–30 mm), indicating that most lesions were small and clinically indolent. Regarding management, 15 patients underwent endoscopic resection alone, 7 were managed with endoscopic surveillance, 3 received somatostatin analogues in combination with endoscopic resection, 2 underwent surgery, and 1 patient received both endoscopic and surgical treatment. During follow-up, recurrence was observed in 4 patients (14.3%). Recurrences occurred in both patients with single and multifocal lesions.

### 3.3 Clinical factors

Dyspeptic symptoms were significantly less frequent in patients who developed T1gNETs compared to those who did not (21.4% vs 43.5%, p = 0.035; OR = 0.35).

When comparing patients with and without T1gNETs, no significant differences were observed in terms of smoking status (3.7% vs 12.3%, p = 0.31), alcohol consumption (22.2% vs 19.9%, p = 0.796) or BMI (25.4 vs 24.4, p = 0.23). No significant associations were found between T1gNETs occurrence and Helicobacter pylori infection or autoimmune comorbidities. Chronic proton pump inhibitor (PPI) use was uncommon in the study population and showed no association with T1gNET occurrence (3.6% in patients with T1gNETs vs 9.5% in patients without T1gNETs).

### 3.4 Serological and biochemical profile

Severe hypergastrinemia (>3× ULN) was more frequent in the T1gNETs group compared to controls (81.2% vs 63%, p = 0.22; OR = 2.54), as well as elevated CgA levels (73.3% vs 53.3%, p = 0.23; OR = 2.36).

### 3.5 Histological associated factors

With regard to histological findings, ECL-cell hyperplasia of the corpus was strongly associated with T1gNETs development (82.1% vs 55.8%, p = 0.0109; OR = 3.65). Corpus intestinal metaplasia was more frequently observed in patients who developed T1gNETs compared to those who did not (92.6% vs 75.3%, p = 0.073; OR = 3.37), although this association did not reach statistical significance. No significant association was found between T1gNETs occurrence and the presence of antral and/or corpus atrophy. Biochemical markers showed a non-significant trend toward association with T1gNETs occurrence.

## 4 Discussion

AIG is a chronic autoimmune disease that leads progressively to destruction of the oxyntic mucosa and subsequently to hypo-achlorhydria. This mechanism progressively contributes to elevate gastrin levels and ECL cells hyperplasia, which leads to increased incidence of T1gNETs (17).

In our population, T1gNETs occurred in 28 out 175 patients (16%), a proportion that appears consistent with previously reported data in patients with AIG and increased compared to general population (approximately 0,001% based on 1/100.000 incidence). Indeed, recent studies have reported a variable incidence of T1gNETs in AIG populations, reflecting differences in study design and follow-up duration. In a large retrospective cohort, Massironi et al. (17) reported that 33 out of 176 patients with histologically confirmed AIG developed T1gNETs during follow-up, corresponding to an annual cumulative incidence of approximately 5.7%. Similarly, Magris et al. (18) observed a prevalence of T1gNETs of 15.4% in a cohort of patients with AIG. Therefore, the prevalence observed in our study falls within the upper range of previously reported data, and may reflect the characteristics of a tertiary referral population, as well as the systematic endoscopic surveillance adopted in our center.

Similarly, the demographic characteristics of AIG patients with T1gNETs in our cohort, including median age and gender, are consistent with recent available data (5, 6, 13, 19, 20).

T1gNETs represent a clinically relevant complication in patients with AIG; however, the identification of patients at highest risk remains challenging. Previous studies have suggested that severe oxyntic atrophy, achlorhydria-related hypergastrinemia, ECL-cell hyperplasia, elevated CgA levels, low pepsinogen I/II ratio, and the presence of intestinal metaplasia may be associated with T1gNETs development (13).

In particular, the progressive loss of oxyntic mucosa and parietal cells, which is a hallmark of AIG, leads to achlorhydria and consequent hypergastrinemia, exerting a trophic effect on ECL cells and promoting their hyperplasia and neoplastic transformation (19, 21). This pathogenetic cascade represents the biological basis for T1gNETs development.

In particular, Magris et al. (18) reported that AIG patients with T1gNETs were characterized by lower pepsinogen I/II ratio and higher gastrin-17 levels, whereas Campana et al. (13) identified CgA >61 U/L, intestinal metaplasia, and male sex as independent risk factors for T1gNETs. Nevertheless, the predictive accuracy of individual biomarkers remains limited, and robust risk-stratification models are still lacking. Therefore, identifying reliable clinical, biochemical, and histological associated factors may help stratify AIG patients and tailor endoscopic surveillance strategies.

In our cohort ECL cells hyperplasia showed a statistical significative correlation with T1gNETs occurrence, these data being in line with literature and reflecting the pathophysiology of this type of NETs (8).

Importantly, most T1gNETs in our cohort were small lesions, with a median size of 4.5 mm, and the majority were successfully managed with endoscopic treatment or surveillance alone. Recurrence was observed in only a minority of patients during follow-up. These findings are consistent with the generally indolent clinical behavior of type 1 gastric neuroendocrine tumors and suggest that, although ECL-cell hyperplasia was associated with tumor occurrence, the detected lesions were predominantly low-risk neoplasms.

According to the study by Vanoli et al, ECL hyperplasia is a risk factor for NET developing and if hyperplasia is severe (i.e., at least 5 confluent micro-nodules and/or nodules involving the lamina propria) there is a higher predictive factor with 92% of sensitivity and 89% of specificity (22).

The association observed between ECL-cell hyperplasia and T1gNET occurrence raises the question of whether histological ECL-cell changes could contribute to a more individualized surveillance strategy in patients with AIG. Although our findings support the role of ECL-cell hyperplasia as a factor associated with T1gNET occurrence, the retrospective design of the study and the limited number of events do not allow definitive conclusions regarding surveillance intervals. Therefore, our data cannot currently support modifications of available guideline recommendations. Nevertheless, as ECL-cell hyperplasia appears to represent a useful marker for identifying patients at increased risk who could benefit from closer monitoring, prospective studies are warranted to validate this hypothesis and develop future risk-adapted surveillance models.

The presence of intestinal metaplasia is a well-established risk factor for gastric epithelial neoplasia, and a modest association has also been reported for T1gNETs. This condition is defined by the replacement of the foveolar and glandular epithelium with intestinal-type epithelium, including goblet and Paneth cells (23). Poveda et al. highlighted that patients with T1gNETs often show advanced “end-stage” metaplasia, corresponding to complete atrophy with mature intestinal differentiation (2).

Similarly, Campana et al. suggested an association between T1gNETs and corpus intestinal metaplasia (13).

In our cohort, intestinal metaplasia showed a positive, although not statistically significant, association with T1gNET occurrence. This finding suggests a potential contributory role of advanced mucosal damage in tumor development, in line with the proposed pathogenetic sequence of AIG. The lack of statistical significance may be related to limited sample size and event rate, rather than the absence of a true biological effect.

Differently from other gastrointestinal NETs, which may present with carcinoid syndrome, gastric NETs are usually non-functioning and often clinically silent. They may occasionally be detected during the diagnostic work-up for anemia, iron deficiency, or vitamin B12 deficiency, while dyspepsia or non-specific abdominal pain are less frequently reported (24, 25).

In a study by Campana et al., including 97 patients with T1gNETs in AIG, diagnosis was incidental in 24.7% of the cases, associated with dyspepsia in 49.5%, and with anemia in 25.8%, without a clear correlation between symptoms and tumor presence (25).

In our cohort, only 6 out of the 28 patients with T1gNETs (21.4%) reported dyspeptic symptoms, confirming that the majority of cases were asymptomatic. This finding is clinically relevant, as it highlights that symptom-based evaluation is not reliable for identifying patients at risk. Indeed, most T1gNETs are incidentally detected during upper endoscopy performed for unrelated indications. Interestingly, in 18 out of 28 patients (64.3%), the diagnosis of AIG and T1gNET was established during the same endoscopic assessment. This finding further supports the frequently silent clinical course of both conditions and underscores the importance of systematic endoscopic and histological evaluation, as a substantial proportion of T1gNETs may already be present at the time of AIG diagnosis.

Another relevant finding of our study is the lack of association between lifestyle factors, including smoking, alcohol consumption, BMI, and T1gNETs occurrence. While Christenson et al. reported a potential association between alcohol use and gNET development, the available evidence on lifestyle factors in this setting remains limited and inconsistent (26).

In this broader context, previous systematic reviews and meta-analyses, have suggested that smoking, alcohol consumption, BMI, and diabetes may play a role in NET development, although results are heterogeneous and often site-specific (27). However, data focusing specifically on T1gNETs in AIG patients are scarce, and previous studies have primarily investigated autoimmune and histological determinants rather than lifestyle-related factors (1, 28).

Therefore, our findings support the hypothesis that, unlike other NET subtypes, lifestyle factors may have a limited or less clearly defined role in the development of T1gNETs, which are more strongly driven by disease-specific mechanisms such as chronic hypergastrinemia and ECL-cell stimulation.

Regarding biochemical markers, both CgA and gastrin levels are typically elevated in patients with T1gNETs. Campana et al. reported that increased CgA levels showed a sensitivity of 61.5% and a specificity of 68.5% for identifying T1gNETs in AIG patients, while Vannella et al. identified CgA as a potential risk factor for tumor development (13, 29).

Indeed, most of the current knowledge on lifestyle-related risk factors derives from studies on neuroendocrine neoplasms as a whole rather than specifically on T1gNETs.

In our cohort, both elevated CgA and gastrin levels were more frequent in patients with T1gNETs, although only with a non-significant trend (respectively 30% vs 25.6%; p=0.22 OR 2.54 and 73.3% vs 53.3%; p=0.23, OR = 2.36 respectively). Overall, the predictive role of CgA appears limited; however, it may still be useful as a marker for treatment monitoring and disease recurrence (30).

Gastrin plays a key role in the pathogenesis of T1gNETs by exerting a trophic effect on ECL cells.

Accordingly, Massironi et al. showed significantly higher gastrin levels in AIG patients who developed.

T1gNETs compared with those who did not (median 992 pg/mL IQR = 449–1500 vs 688 pg/mL IQR = 423– 1200, P = 0.03), with sensitivity and specificity of 90.9% and 1.4%, respectively and an overall diagnostic accuracy of 30% (17). Similar findings were reported by Jové et al., who observed markedly increased gastrin levels in patients with gNETs (31). Conversely, Li et al. did not find significant differences between groups (19). Also in our results no statistically significant difference has been found in gastrin levels (81.2% vs 63.0%, p=0,2261; OR = 2.54), suggesting that, although hypergastrinemia is central to the pathogenesis of T1gNETs, its diagnostic performance in predicting tumor occurrence is limited. Indeed, gastrin levels are typically elevated in most patients with AIG due to achlorhydria, thereby reducing their specificity as a biomarker for identifying patients at higher risk of developing T1gNETs. A potential confounding effect of chronic proton pump inhibitor (PPI) therapy should also be considered, as long-term PPI exposure may induce secondary hypergastrinemia and ECL-cell hyperplasia. However, in our cohort, chronic PPI use was uncommon and was not more frequently observed among patients who developed T1gNETs (3.6% vs 9.5%), suggesting that PPI exposure is unlikely to explain the association observed between ECL-cell hyperplasia and T1gNET occurrence.

According to current guidelines, patients with AIG require regular endoscopic surveillance due to their increased risk of gastric neoplasia, including both adenocarcinoma and NETs. The MAPS II guidelines (Management of Epithelial Precancerous Conditions and Lesions in the Stomach) previously recommended endoscopic follow-up every 3–5 years in patients with advanced atrophic gastritis (32).

More recently, updated ESGE guidelines have suggested a more standardized surveillance interval of every 3 years in patients with AIG, emphasizing the need for high-quality endoscopic assessment with systematic biopsies (11).

In this context, our findings, showing a relatively high incidence of T1gNETs asymptomatic patients, further support the importance of regular endoscopic surveillance in this population, as clinical symptoms alone are not sufficient to identify patients at risk.

It should be noted that this study is subject to certain limitations, including its retrospective nature. Furthermore, in some cases, the AIG diagnosis was made in other centers, preventing a reliable assessment of disease duration before referral. In addition, in a substantial proportion of patients, AIG and T1gNET were diagnosed concomitantly during the same endoscopic evaluation, making it difficult to accurately investigate the temporal relationship between the two conditions. Future multicenter studies with population-based sampling are required to validate these findings. Nevertheless, the present study offers a valuable real-world analysis of AIG and occurrence of T1gNETs in a large patient cohort in a tertiary care center. Unlike most previous studies, which have primarily focused on selected histological or biochemical determinants, our study simultaneously evaluated clinical presentation, lifestyle-related variables, biochemical markers, and histological features within the same well-characterized AIG cohort. This comprehensive approach provides a broader real-world assessment of factors associated with T1gNET occurrence and may contribute to the development of future risk-stratification models.

## 5 Conclusions

This study investigated the clinical, biochemical and histological data of patients with AIG with and without T1gNETs. Our results confirm an association between ECL hyperplasia and T1gNETs occurrence and only a trend with corpus metaplasia. Of note, an inverse statistic significant correlation with dyspeptic symptoms was seen. These findings, even though preliminary, may help in the risk stratification of patients with AIG, but further validation for their potential clinical utility in T1gNETs screening and surveillance is warranted.

## Data Availability

The raw data supporting the conclusions of this article will be made available by the authors, without undue reservation.
